# A Comprehensive Integrative Review of the Factors Associated with Spontaneous Preterm Birth, Its Prevention and Prediction, Including Metabolomic Markers

**DOI:** 10.1055/s-0040-1701462

**Published:** 2020-01

**Authors:** Renato Teixeira Souza, José Guilherme Cecatti

**Affiliations:** 1 Department of Obstetrics and Gynecology, School of Medical Sciences, Universidade Estadual de Campinas, Campinas, SP, Brazil

**Keywords:** preterm labor, premature rupture of fetal membranes, risk factors, screening programs, perinatology, metabolomics, trabalho de parto prematuro, ruptura prematura de membranas fetais, fatores de risco, programas de rastreamento, perinatologia, metabolômica

## Abstract

Preterm birth is a major maternal complication that has a great impact on perinatal and neonatal health, with consequences suffered during childhood and adulthood. Little is known about its etiology and development, resulting in poor screening, prediction and preventive methods. The present integrative review discusses the current knowledge regarding some risk factors for preterm birth, the differences between screening and prediction methods, the limitations of some current preventive interventions, the importance of applying standardized concepts for exposures and outcomes, and why it is important to develop more accurate and reproducible methods to predict preterm birth. In addition, the authors introduce the concept of metabolomics and the technology involved in this technique, and discuss about how it has become a promising approach to identify biomarkers for spontaneous preterm birth.

## Resumo

Parto prematuro é uma complicação obstétrica de grande impacto para saúde perinatal e neonatal, tendo consequências também para a infância e a vida adulta. Pouco se sabe sobre sua etiologia e fatores determinantes, o que limita os métodos de rastreamento, predição e prevenção. Esta revisão integrativa traz a discussão sobre o conhecimento atual sobre fatores de risco para parto prematuro espontâneo, as diferenças entre métodos de rastreamento e predição, as limitações das atuais intervenções preventivas, a importância de se aplicar conceitos padronizados para exposição e desfecho na investigação de parto prematuro espontâneo, e porque é importante desenvolver métodos precisos e reprodutíveis para predizer o parto prematuro. Por fim, introduzimos o conceito de metabolômica e da tecnologia envolvida nessa técnica, e discutimos como ela tem se mostrado uma abordagem prosmissora para identificar biomarcadores associados ao parto prematuro espontâneo.

## Introduction

## Definition and Impact of Prematurity

The birth of a preterm baby may have diverse negative consequences for the baby him/herself, (his or her) neonatal life, childhood and adulthood, the family, the healthcare system/service, and society as a whole. The present manuscript focuses on the factors associated with preterm birth, the perinatal outcomes and the ways to predict them, supported by the hypothesis that it is possible to better understand and predict the preterm birth process, creating opportunities for increased effectiveness in the prevention of this condition.

It took several decades to consolidate the definition of preterm birth. At the beginning of the 20th century, newborns weighing less than 2,500 g at birth were considered preterm, based primarily on neonatal behavior and progression to neonatal mortality, intracranial hemorrhage and other morbid conditions.^1^ In 1950, a group of experts from the World Health Organization (WHO) published a technical report^2^ defining preterm newborns as those weighing less than 2,500 g at birth, or those born before 37 weeks of gestation. In this document, the WHO established two priorities for the promotion of research and specific programs aimed at minimizing the consequences of preterm birth: prevention and preterm infant care.

Preterm birth may be classified according to the clinical presentation or the motivator: spontaneous, when due to spontaneous preterm labor (contractions, cervical effacement and dilatation) or preterm premature rupture of the membranes (P-PROM); and therapeutic, when theoretically there is a condition that poses a risk to the mother and/or the fetus, generating sufficient motivation for resolution at a preterm gestational age.^3^ Furthermore, iatrogenic preterm birth is defined as birth due to therapeutic intervention without the existence of sufficient risks to justify any intervention, that is, due to convenience, maternal desire or simply without scientific evidence for premature resolution.^4^ For each of the three subtypes of preterm births (spontaneous preterm labor, P-PROM, or therapeutic) there are different risk factors and maternal and perinatal associated outcomes.^5^ ^6^ ^7^

Therefore, at least the distinction between spontaneous and therapeutic preterm birth is highly recommended when studying the determinants and consequences of preterm birth. The recognition that not every preterm birth occurs because of the same determinants was an early step to study the causes and develop preventive strategies. Preterm birth is also categorized according to gestational age at birth, and is divided into: late preterm (between 34 weeks + 0 days and 36 weeks + 6 days), moderately preterm (from 32 + 0 weeks to 33 + 6 weeks), very preterm (from 28 + 0 weeks to 31 + 6 weeks) and extremely preterm (< 28 weeks).^4^ ^8^

Pregnancy of a singleton or multiple fetuses (twins) confers great differences not only in terms of the incidence of preterm birth, but also concerning its associated factors and the maternal and perinatal outcomes.^9^ A study evaluating offical data of the Brazilian System of Information on Live Births (Sinasc, in Portuguese) from 2011 to 2014 shows that ∼ 53% of twin pregnancies progress to preterm deliveries.^10^ Furthermore, there is an increase in complications, such as maternal near-miss events, maternal mortality, perinatal mortality, preeclampsia, and postpartum hemorrhage.^9^ ^10^ ^11^ The increased incidence of complications due to multiple pregnancies associated with a higher rate of twin pregnancies in the last decades denotes the importance of this type of pregnancy in preterm birth and maternal and perinatal health.^9^ ^12^ Twin pregnancies are not the focus of the present review, since an adequately designed and appropriate approach would be required for this type of pregnancy in order to evaluate its associated factors, preventive and predictive methods for preterm birth, and the respective perinatal outcomes.^11^

A study^13^ by the World Health Organization (WHO) estimated that around 15 million preterm births occur annually worldwide, representing a rate of 10.3% of all deliveries.^3^ International data from 1990 to 2010 from 65 countries of Europe, Australasia, and the Americas showed that the absolute number of preterm births and preterm infant rates increased during this period.^3^ Countries from North Africa, Sub-Saharan Africa and Asia represent little more than 70% of deliveries and 80% of preterm births across the world. Furthermore, only five countries – India, China, Nigeria, Bangladesh, and Indonesia – account for almost half of preterm births worldwide.^13^ Around 17% of preterm births occur in the Americas (North, Central and South America, and the Caribbean), Europe and Australia. However, these regions have the highest proportion of extreme preterm births.^13^ Preterm birth represents a huge public health issue in all contexts and countries, be them low-income or high-income.^14^ ^15^

Complications due to preterm births account for more than 1/3 of neonatal deaths worldwide, representing over 1 million newborn infants who died in the first month of life in 2010. The impact of complications due to preterm birth also has repercussions regarding childhood health indicators. It is the second cause of death until age 5 globally, and the first cause of death in mid-income and high-income countries.^3^

Since the 1950s, many advances have been made in the number of options and in the level of scientific evidence-based preventive measures for neonatal complications due to preterm birth. Among those are measures of tertiary prevention, such as the use of tocolytics and corticotherapy for the prevention of hyaline membrane, intraventricular hemorrhage and necrotizing enterocolitis; magnesium sulfate for the prevention of cerebral palsy in cases of imminient preterm delivery; and antibiotic therapy for the prevention of neonatal sepsis and to prolong the latent phase in cases of P-PROM.^16^ ^17^ ^18^ ^19^ ^20^ Although these measures have a short- and long-term impact on perinatal morbidity and mortality, they are usually only adopted when the preterm birth has already begun and its ocurrence is imminent. Earlier identification of these cases still in the asymptomatic phase could theoretically increase the window of opportunity for preventive interventions and bring about better perinatal outcomes.^21^ ^22^

There were also advances in the identification and institution of early therapies for neonatal complications such as neonatal sepsis, hypothermia, visual, cerebral (intra- and periventricular hemorrhage), auditory and/or neuropsychomotor impairments, providing the newborn with the possibility of earlier neonatal follow-up and better long-term results.^23^ ^24^ ^25^ The advent of continuous positive airway pressure (CPAP), for example, mechanical ventilation, the use of exogenous surfactant in the 1970s, and refinement of oxygen saturation targets in neonatal oxygen therapy in the last decade have resulted in significant improvement regarding neonatal survival, especially for extremely preterm infants.^23^ Advances in tertiary and quaternary preventions, which correspond to a decrease in complications or adverse events after the emergence of a disease or its sequelae, do not seem to be equally accompanied by primary or secondary interventions. The difficulty is due, in part, to the lack of knowledge of the pathophysiology of preterm birth and its risk factors, which limits the development of preventive measures and effective prediction models.

## Risk Factors for Preterm Birth and Prediction

Risk factor is a term used to designate conditions, characteristics, habits or markers that, when present, increase the probability of occurrence of a specific injury. Risk, therefore, is related to the onset of a condition.^26^ ^27^ Gender, ethnicity and age are considered fixed risk factors, and weight, body mass index (BMI), smoking, alcoholism or use of a condom, for example, are modifiable risk factors.^26^ They may have different strengths of association with the risk of developing a determined condition depending on the combination of other factors, such as time of exposure or even the population studied.^27^ ^28^

An example of a combination of factors is BMI and gestational weight gain. The National Academy of Medicine (NAM), in the United States, formerly known as the Institute of Medicine (IoM), categorized BMI into low weight (BMI < 18.5 kg/m^2^), normal (BMI between 18.5 kg/m^2^ and 24.9 kg/m^2^), overweight (BMI between 25.00 kg/m^2^ and 29.9 kg/m^2^) and obesity (BMI ≥ 30.0 kg/m^2^).^29^ A study^30^ evaluating data from a prospective cohort with more than 45 thousand American pregnant women showed that BMI and gestational weight gain seem to have different impacts on the risk of having different subtypes of preterm birth, depending on the category of the initial BMI and the respective weight gain. Nevertheless, in this study, gestational weight gain was calculated by subtracting the initial weight from the last weight before childbirth. This method does not consider that women with preterm delivery had less weeks of gestation to gain weight, mainly in the third trimester, the period in which highest rate of weight gain occurs according to the IoM.^29^ This results in a biased comparison of weight gain, for example, between a woman delivering at 28 weeks and another woman delivering at 41 weeks. In this case, the use of weight gain rate per week would be highly recommended. A systematic review^31^ evaluating 39 studies including data on almost 1.8 million women highlights the lack of homogeneity in categorizing the initial BMI and defining the outcome according to the subtypes of prematurity and the estimate of the gestational age. Studies with the goal of circumventing these limitations are still scarce, although necessary in order to better understand the role of BMI and gestational weight gain in the risk of the occurrence of different subtypes of prematurity.

Didactically, the risk factors for preterm delivery may be classified as clinical, semiological, microbiological, ultrasonographic and biochemical.^32^ Enviromental, social and genetic factors are also included.^33^ According to some systematic reviews,^3^ ^7^ ^33^ the main clinical risk factors for preterm birth, that is, those that have a higher independent association with preterm delivery, are history of previous preterm delivery, smoking, and multiple pregnancy. A history of previous preterm birth is the most important risk factor for preterm birth. A previous preterm delivery increases 3- to 4-fold the risk of having a new preterm delivery.^7^ ^34^ ^35^ ^36^ Theres is a relative amount of information available, since it is collected from the basic obstetric clinical history, preferentially detailing how and at which gestational age the preterm birth occurred.^34^ ^35^ ^36^ The earlier the preterm delivery, the higher the risk of having a new case of preterm delivery; the number of recurrences was also associated with a 5- to 6-fold increase in the chance of having a new preterm delivery.^36^ However, a limitation of this risk marker is that it cannot be applied to nulliparous women.

Smoking is a modifiable risk factor associated with an incidence of preterm birth that is 3- to 4-fold higher in smokers than in non-smokers.^33^ ^37^ The risk seems to be dose-dependent: the more cigarettes smoked, the higher the risk. In addition, the risk is also associated with passive smokers, that is, pregnant women exposed to cigarette smoke.^38^ ^39^

It is important to emphasize that a condition that is associated with an outcome may not always be considered a risk factor for the condition. Exposure prior to the appearance of a disease, its removal or reduction is a characteristic associated with a lower incidence of disease; dose-dependence and measure of exposure need to be considered in the relation of risks. These characteristics are preponderant in the application of risk factors as predictors.

The great challenge lies in the limited knowledge of the patophysiology and etiology of preterm birth. There are some propositions concerning the mechanisms involved in preterm birth. A hypothesis by Behrman and Butler^40^ highlighted the role of uterine distension, decidual hemorrhage or thrombosis, inflammatory or infectious processes, activation of the hypothalamus-pituitary-adrenal axis and stress, which, alone or in conjunction, may lead to pro-inflammatory activation of the decidua and membranes. Prostaglandins and metalloproteinases, in turn, along with other inflammatory agents, may promote cervical remodeling and/or uterine contractions, leading ultimately to preterm labor and/or P-PROM.^40^ In contrast, Menon^41^ categorized the risk factors as static and dynamic, also proposing a complex and not fully clear interaction between diverse inflammatory, immunological, environmental, and epigenetic mechanisms, among others, that culminate in senescence and “weakening” of amniotic membranes, decidual and myometrial activation, cervical effacement, and, finally, preterm birth. Multiple markers involved in these mechanisms are studied as potential predictors of preterm birth.

Systematic reviews have identified many studies evaluating these different biological and biophysical markers, highlighting the fetal fibronectin l (fFN) and the phosphorylated isoform of insulin-like growth factor binding protein (phIGFBP-1), binding proteins present between the chorion of the amniotic membrane and the maternal decidua, and cervical length measurement in the second trimester of pregnancy by transvaginal ultrasound. Systematic reviews have concluded that those markers are not sufficiently accurate to be useful in the clinical prediction of preterm birth, especially in asymptomatic women.^32^ ^42^ ^43^ ^44^

A Dutch prospective cohort study^45^ including nearly 12 thousand women assessed the performance of cervical length measurement in the prediction of preterm birth. The measurement of the cervix was performed between 16 and 22 weeks of gestation. It was shown to be poor and did not vary significantly between nulliparous and multiparous women, as well as among women considered to be at low or high risk. The area under the receiver operating characteristic (ROC) curve ranged from 0.56 to 0.61 for the multiparous and the low-risk nulliparous groups respectively, that is, the method fails to identify around 40% to 50% of women who will have a preterm delivery.

Fetal fibronectin in the vaginal secretion does not show much superior results in asymptomatic women. A cohort study^46^ from the United Kingdom analyzed the performance of fFN collected from the cervicovaginal secretion as a predictor of spontaneous preterm delivery at less than 34 weeks of gestation. Almost 1,500 women were included, and the vaginal secretion was collected from 22 to 28 weeks. The study showed that levels above 50 ng/ml have a sensitivity of 46.5% and a specificity of 88.7%. The higher the cut-off point for fFN in the vaginal secretion, the higher the negative predictive value (NPV) and specificity of the fFN. When the cut-off point was 500 ng/ml, the specificity and NPV were higher than 90%. However, its clinical application is still limited, since it is expected that a large part of the population does not have such high levels of fFN during this phase of gestation, and the test has a very low sensitivity with this cut-off point, that is, many women with a preterm delivery do not achieve such high fFN levels in the vaginal secretion during this period.

Other propositions have attempted to address the association between multiple factors involved in the development of preterm birth. A group of experts^4^ ^47^ proposed a classification of women at risk for preterm delivery, according to phenotypes. Empirically, those authors defined that the development of preterm birth is not exclusive to a single group of women who necessarily have similar characteristics and risk factors. On the contrary, probably different groups of women have conditions in common that are associated with preterm birth and its different subtypes. Conditions that potentially define the phenotypes of preterm birth were divided into maternal, fetal and placental conditions. These conditions are not based on risk factors, but depend on conditions present in the index pregnancy that determine the occurrence of preterm birth. The application of this new classification could help understand the associations between the determinants of preterm birth, help measure the benefits of preventive measures, help identify conditions most impacted by these measures, and, ultimately, help physicians understand the subgroups of women that are at higher risk of having different subtypes of preterm birth.

The aforementioned group of experts, with the aid of other collaborators, applied this concept through a secondary analysis of an international multicenter cohort study named INTERGROWTH 21st.^48^ Slightly more than 50 thousand women had estimates of gestational age calculated by obstetric ultrasound, and 5,828 women had preterm deliveries (10.5%). A cluster analysis of preterm births was conducted, grouped or not, according to one or more of six maternal conditions, seven fetal conditions and three placental conditions. Finally, 12 clusters were identified, drawing attention to cluster 1, in which 1.747 (30%) women had none of the 16 predefined conditions. Over 80% of women from this cluster had preterm births either due to preterm labor or P-PROM. On the other hand, the majority of women were divided into 11 clusters, which were charaterized by major conditions such as preeclampsia/eclampsia, chorioamnionitis, twin pregnancies or bleeding at the beginning of pregnancy etc., showing that it is possible to identify determining factors in subgroups of women with preterm birth, which helps physicians to understand the etiology and identify women at higher risk. Nevertheless, this concept still requires reproducibility. Validation of the cluster determination, along with the predefining conditions in other populations, is necessary. Thus, we are faced with the need to better explore risk models for preterm birth, to identify risk factors and their associations, in order to determine the etiological theories and develop models that are efficient at predicting spontaneous preterm birth.

## Prevention of Preterm Birth

According to Geoffrey Rose,^28^ there are two prevention strategies: one based on individual preventive measures through the identification of individuals at higher risk of developing the condition; and the other based on measures of the general population, irrespective of the existence of risk factors. Available access to prenatal care, qualified childbirth and postpartum care, incentive programs for healthy lifestyle habits and protection of a woman's right to health care are important strategies that may have an impact on maternal and perinatal health indicators, including preterm birth.^49^ A good example of exposure that has preventive measures based on both strategies is smoking. Around 50% of American pregnant women stop smoking in the first trimester of pregnancy.^50^ Individual policies such as counseling, stimulation of pharmacologic replacement of nicotine, psychological support, and even financial incentives have an impact on the prevention of adverse perinatal outcomes. Governmental policies such as dissociating the image of the cigarette as a healthy and socially desirable habit through campaigns in the media, increase in taxes for the tobacco industry, and laws that restrict areas where smoking is allowed also demonstrated a beneficial effect.^50^ A systematic review^50^ including clinical trials testing different strategies for cessation of smoking showed that interventions reduced preterm births by ∼ 15%. Although continuous effort and specific public policies are necessary, this is a good example of how identifying the risk associated with prevention strategies may result in more cost-effective and better maternal and perinatal outcomes.^50^ ^51^ ^52^

The identification of factors associated with a higher risk of developing spontaneous preterm birth may be useful to help physicians understand its pathophysiology and identify women at higher risk who might benefit from prevention strategies. In the latter case, it may also possible to distinguish between screening for risk and prediction of preterm birth. Although both methods use risk factors as the basis for their models or algorithms, their practical application may be quite distinct.^53^

For example, women with transvaginal ultrasound assessment of cervical length between 20 mm and 25 mm, which was measured in the second trimester by a standardized technique, had an incidence of preterm birth ranging from 22% to 32%.^54^ ^55^ This incidence may reach 56% in cases of cervical length shorter than 5 mm.^55^ The increased incidence in women with a cervix shorter than 25 mm, in comparison to the general population, confers a 4- to 5-fold higher risk of having a preterm birth. Observational studies^45^ ^54^ ^55^ in different populations confirm this inverse association between uterine cervix measurement in the second trimester and the prevalence of spontaneous preterm birth. Therefore, uterine cervices shorter than than 25 mm were considered “short”, and those longer than 25 mm were considered “normal”.^45^ ^54^ Based on the uterine cervix measurement to stratify women at higher risk, several clinical trials^56^ ^57^ have tested preventive interventions for spontaneous preterm birth and its association with adverse perinatal events, initially comparing natural micronized progesterone (vaginal tablet) or hydroxyprogesterone caproate (intramuscular injection) with placebo. Systematic reviews with meta-analysis^56^ ^57^ showed that the use of vaginal progesterone seems to be beneficial for the reduction of preterm birth before 37, 34 and 28 weeks and of neonatal morbid conditions. However, differences in reduction rates of different morbid conditions or even preterm birth may be attributed to different selection criteria for women included in clinical trials.

The OPPTIMUM study,^58^ for example, a British multicenter study including 65 centers in the United Kingdom and 1 in Sweden, published in 2016 (after the systematic review), aimed to evaluate not only the benefit of progesterone in reducing prematurity and neonatal morbidity, but also its long-term effect on the child. The study selected women with singleton pregnancies at high risk of having preterm birth based on: history of previous preterm birth, gestational loss in the second trimester, P-PROM, cervical procedure, and positive vaginal fetal fibronectin. A year after the begining of the clinical trial, the researchers decided to include women at “mid-high” risk, which was defined as women with negative fetal fibronectin, but with a history of spontaneous preterm birth at before 34 weeks, or uterine cervix shorter than 25mm in the second trimester. This double-blinded controlled study randomized more than 600 women in each group (vaginal progesterone 200 mg versus placebo), and demonstrated that progesterone was not beneficial in reducing preterm birth or the majority of perinatal morbid conditions, such as pulmonary bronchodysplasia, neonatal infection, necrotizing enterocolitis, and neurological development and neurocognitive score at 2 years of age. However, the study showed a reduction in neonatal death (non-adjusted odds ratio [OR] of 0.17 [0.06–0.49], *p*-value of 0.0009) and in cerebral alterations on ultrasound (non-adjusted OR of 0.50 [0.31–0.84], *p*-value of 0.008). The authors of this study concluded that the subgroups of women who might benefit from progesterone are not easily identified by the current screening strategies. This should encourage studies on new prevention strategies as well as those aimed at identifying women that may be potentially eligible to undergo this treatment.

Another technique that has been studied for decades is cerclage, which is primarily based on suture of the uterine cervix or isthmus-cervical region to prevent early effacement/dilatation of the cervix. The Shirodkar^59^ technique, described in 1953, and a technique by McDonald^60^ in 1957 are the basis for all of the subsequently described variations. These techniques were initially proposed for cases with a history of cervical insufficiency, a known cause of late abortion and extreme prematurity. A Cochrane systematic review of 15 clinical trials showed advantages of these techniques in prolonging pregnancy, decreasing the rates of neonatal morbidity and prematurity when indicated to women with a history of cervical insufficiency.^61^ The advent of the cervical measurement in the second trimester, associated with a history of preterm birth, seems to have improved the identification of women who will benefit from cerclage to prevent preterm birth, particularly in cases in which there is still no history of recurrent pregnancy loss.^62^ This shows that the search for an association of risk factors in the prevention of preterm birth may still be very useful, even in situations in which a good solution was apparently found, as for cervical incompetence and cerclage.

It is also worth mentioning that another intervention was studied for the prevention of preterm birth in high-risk women. A pessary, a device made of firm silicone in the shape of a convex ring, is inserted into the posterior vaginal fornix and fastened to the cervix. The theoretical mechanism for the prevention of preterm birth is based on: 1) a change in the axis of forces resulting from the uterine body and isthmus that act on the cervix; and 2) a potential closure of the cervix with consequent strengthening of the cervical canal and the immunologic barrier of the cervix, preserving the amniotic membranes from contact with the vaginal environment.^63^ Although the subject has been studied since the middle of the 20th century, the identification of women who actually benefit from this intervention remains a challenge. The Pesario Cervical para Evitar Prematuridad (PECEP, “Cervical Pessary to Prevent Prematurity”) study trial,^63^ published in 2012, was the first randomized study using the pessary (*versus* expectant management) to prevent preterm birth. Selecting pregnant women at high risk based on cervical length measurement in the second trimester, with slightly more than 190 women per group, the study showed that the incidence of preterm births before 34 weeks decreased by 80%. Subsequent studies demonstrated conflicting results, and did not confirm such a reduction in the incidence of preterm births observed by Goya et al.^63^ However, the selection of eligible women and the association with other interventions, such as progesterone, is heterogenous among studies.^64^ ^65^ ^66^ ^67^

Despite the advances/benefits resulting from a combination of screening for risk and interventions, such as, progesterone, the pessary and cerclage in women selected based on risk factors, there still seem to exist limitations and heterogeneity in screening. Better results from the use of these measures may be potentially hindered. Which women might actually benefit from the use of progesterone during prenatal care? Which women might not benefit from any preventive intervention? Furthermore, there is no consensus over which level of risk estimate determines that a woman should in fact be considered at high risk. Improved identification of women at high (or low) risk of having a preterm birth with the development of prediction models that have good discriminatory performance may be quite relevant to advance the investigation of the benefits of using (or not) progesterone, the pessary or any other form of preterm birth prevention.

## Risk Assessment and Prediction of Preterm Birth

The description of these prevention studies and their interpretations are important to highlight the fundamental role of the adequate screening of women who may benefit from prevention strategies. Distinctions must be made regarding the risk assessment model and a predictor model for an outcome. This distinction may actually help physicians understand the clinical application of a screening strategy for women at high risk of having a preterm delivery.

As an example of a risk marker, the cervix is known to be independently associated with a higher risk of having a preterm birth.^54^ ^55^ Although this may be useful for the implementation of differentiated care, suggesting screening and interventions for the subgroup of women with a short cervix, this practice is fragile in terms of the population, and has a low impact on prevention.^68^ The reason for this is that, despite a higher risk of having a preterm birth, a woman with a short cervix has the highest odds of having a term birth. Furthermore, the shortening process of the cervix may not occur early in the recommended screening phase (the second trimester, between 18 and 24 weeks). In summary, the cervix is a marker of low sensitivity (a considerable proportion of women with a short cervix are likely to deliver at term). At the same time, the marker has a low rate in the general population, since cervices measuring 25 mm and 20 mm correspond respectively to a 5th and a 3rd percentiles in the population curve of cervical measurement.^54^ A cohort^54^ with almost 3 thousand pregnant women evaluated the performance of 28 markers in the second trimester of pregnancy, and it showed that a short cervix has a sensitivity of 36.8% for preterm birth before 35 weeks. This means that almost two-thirds of women with preterm birth below this gestational age would not be screened using this criterion, resulting in elevated false-negative rates of the method. Therefore, despite the positive association with preterm birth, a short cervix seems to be an inappropriate marker to compose predictive models, resulting in low efficacy when employed in the clinical practice.^45^ ^54^ ^55^ Even serial measurements of the cervix, based on the theory that shortening of the cervix over the weeks could be a better predictor of preterm birth, showed a worse predicitive performance than a single measurement.^69^

A prospective observational study^70^ included more than 9 thousand nulliparous pregnant women from 8 centers across the United States, and it evaluated the performance of fetal fibronectin and transvaginal measurement of the uterine cervix in predicting spontaneous preterm birth. The area under the ROC curve was of 0.59 for fetal fibronectin ≥ 50 ng/dL, and of 0.67 for cervices shorter than 25 mm. The model containing both variables had an area under the ROC curve of 0.67. The authors concluded that the performance was poor and of low clinical utility.

In summary, systematic reviews have concluded that there are no markers in the literature that can be applied in the clinical practice to predict spontaneous preterm birth with a good performance, and that enable new preventive approaches and studies in this area.^71^

## Metabolomics and Preterm Birth

The term “omics sciences” is applied to the field of knowledge that focuses on genomic studies, gene identification, DNA sequence polymorphisms, genes and the genome; transcriptomics, which is focused on the study of gene expression – RNAs; proteomics, the large-scale study of proteins; and metabolomics, the scientific study of chemical processes involving metabolites.^72^ ^73^ ^74^ ^75^ The application of each technique to investigate markers or the pathophysiology of diseases, primarily those involving complex mechanisms that have not yet been fully elucidated, is basically dependent on the objectives and resources available. Actually, an integrated application of the various methods may be the best option.^74^ The main advantage of metabolomics is that it seems to be closer to disease phenotype, presenting the result of the final pathway of interactions between genes, RNAm and proteins. According to Dettmer et al,^76^ genomics tells what can happen, transcriptomics, what appears to be happening, proteomics, what makes it happen, and metabolomics, what has happened and what is happening.

Metabolomics is the science that studies metabolites, small molecules present in different chains of the metabolism of an organism.^77^ These small molecules may be substrates, products and cofactors of intracellular and extracellular chemical reactions such as aminoacids, biliary acids, carbohydrates, lipids, vitamins and others.^78^ The group of metabolites in a certain sample or organism is called metabolome. Different techniques are used to identify and quantify metabolites, such as mass spectrometry coupled with liquid or gas chromatography or magnetic resonance imaging. Furthermore, diverse configurations or variants may be used to obtain a better performance, depending on the metabolite of interest, its polarity, the mass spectrum to be studied, or other physicochemical characteristics of the metabolites and samples to be analyzed. Technological advances in instruments for data acquisition and bioinformatics have provided sufficient aid, so that metabolomics is able to identify and analyze hundreds or even millions of metabolites in a certain biological sample. Studies on diverse applications in biological samples demonstrate a high sensitivity in the detection and measurement of metabolites.^77^

By identifying and quantifying metabolites, this technique is capable of showing the fingerprint of the metabolic interactions of the organism in a certain sample at a certain time. Metabolomics is a technique known as hypothesis-free, that is, it does not require an initial hypothesis. Instead of testing a certain hypothesis, the technique may generate novel hypotheses through its results when elucidating the markers and biological pathways involved in the process of disease development, which may not have been clarified.^74^ ^76^ ^77^ It may be a relevant complementary tool for the construction of knowledge in diseases in which the pathophysiology has yet to be fully elucidated and possibly involves multiple complex genetic and environmental interactions, such as preterm delivery, preeclampsia and fetal growth restriction.^77^ ^78^

Metabolomics has been applied in biological samples for the investigation of processes ranging from embryogenesis to the emergence of complex diseases such as cancer, Parkinson disease, diabetes and depression.^79^ In the area of maternal and perinatal health care, it has been mainly applied to identify biomarkers, which are clinically useful for the performance of diagnostic or prognostic predictions.^74^ ^78^ ^80^

After identifying 45 metabolites significantly associated with preclampsia in serum samples collected at 15 weeks of gestation from a group of ∼ 39 nulliparous pregnant women with a history of preeclampsia (compared with 40 pregnant women without complications), 14 metabolites were selected to compose the final model (validation). The model resulted in an area under the ROC curve of 0.92, and an OR of 23 (95% confidence interval [95%CI]: 7–73).^81^ Another study using samples of a similar number of women who progressed to preeclampsia, as well as samples collected a short time earlier (between 11 and 14 weeks), showed more modest results, albeit still promising. The model containing 4 metabolites has a detection rate of only 50%, assuming a false-positive rate of 10%, with an area under the ROC curve of 0.81 for cases of preeclampsia.^82^ Fews studies on the identification of biomarkers to compose prediction models for preterm birth have been published until now, and some narrative reviews of the subject have described a great heterogeneity in the methodology employed.^74^ ^78^ ^80^ To date, there are no systematic reviews that analyze the performance of metabolomics in predicting spontaneous preterm delivery.

Many aspects regarding the most effective method to investigate preterm birth using metabolomic markers should be discussed. First, there is the type of sample used (urine, blood, amniotic fluid, hair, vaginal secretion). Then, there is the time for sample collection (during the clinical presentation of preterm birth or in the early phase of pregnancy, when there are no symptoms). Furthermore, metabolomics demands a high methodological rigor in the collection and storage of biosamples, since this is a highly sensitive method to identify small low-weight molecules; various types of “noise”, or interference in data acquisition, may hinder the identification of these molecules. Heterogeneity in sample collection and storage may be the cause. In addition, a well-delineated study design, with well-defined outcomes, following clear classifications, associated with sequential validations of findings is crucial for the reliability and reproducibility of this technique. Finally, still in the phase of data analysis, caution regarding some important considerations is emphasized. For example, hundreds or even thousands of metabolites are usually analyzed at the same time in a sample. Since the number of variables (metabolites) is much higher than the number of samples (individuals), the analysis is very susceptible to significantly false results. To correct this effect, the Bonferroni correction may be used, attenuating the significance of the *p*-value according to the number of variables (metabolites) analyzed. The false discovery rate (FDR) method may also be applied to control the number of false-positive conclusions, and significance is only assigned to the “most promising” variables.^83^ This technique was proposed by Benjamini and Hochberg^83^ in the 1990s, and it is based on the proportion between the true null hypothesis (H0) and the rejected null hypothesis, decreasing the possibility of markers considered to be statistically discriminatory. Actually, these markers are not discriminatory. These are only two examples of methodological care required in the phase of data analysis.

Many questions need to be answered concerning the mechanisms involved in the development of preterm birth: why do some women have early cervical remodeling (with an evident short cervix on the transvaginal ultrasound in the second trimester) and others do not? Which and how are the interactions between different risk factors, including infection, vaginal bleeding and body mass index, and how can they determine preterm birth? In theory, metabolomics depicts the final pathway resulting from these interactions, and it seems to be a useful approach not only to predict spontaneous preterm birth, but also to elucidate the many mechanisms involved.

## Conclusion

In order to adequately address the investigation of preterm birth, its associated factors and perinatal outcomes, a robust methodological approach is required, using judicious and standardized definitions of exposures and outcomes. Based on this premise, a multifaceted comprehensive approach, albeit integrated, was proposed for data exploration on the factors associated with preterm birth, its prediction and the perinatal outcomes, which may be capable of generating new knowledge regarding this issue. It is expected that the results of this approach may contribute to the prediction of the most effective performance and better understanding of the factors associated with spontaneous preterm birth and consequent adverse perinatal results, collaborating with the development and application of public policies to prevent preterm birth and its perinatal consequences. We acknowledge the fact that the present is an integrative review based on a biased search in the literature and on the interpretation of the studies and respective findings. Although we have not used the standard tools and strategies to measure and report these biases, we consider that it is a great opportunity to raise the discussion about some of the risk factors associated with sponteaneous preterm birth, how preventive strategies based on these factors have been implemented, and the results so far. We expect that, despite the limitations of the present integrative review, it may contribute to the discussion about recognizing women at a higher risk of having sponteaneous preterm birth and how to prevent it.
